# The crystal structure of the catalytic domain of a eukaryotic guanylate cyclase

**DOI:** 10.1186/1472-6807-8-42

**Published:** 2008-10-07

**Authors:** Jonathan A Winger, Emily R Derbyshire, Meindert H Lamers, Michael A Marletta, John Kuriyan

**Affiliations:** 1 Department of Molecular and Cell Biology, University of California, Berkeley, CA, USA; 2 Department of Chemistry, University of California, Berkeley, CA, USA; 3 Howard Hughes Medical Institute, University of California, Berkeley, CA, USA; 4 Division of Physical Biosciences, Lawrence Berkeley National Lab, Berkeley, CA, USA

## Abstract

**Background:**

Soluble guanylate cyclases generate cyclic GMP when bound to nitric oxide, thereby linking nitric oxide levels to the control of processes such as vascular homeostasis and neurotransmission. The guanylate cyclase catalytic module, for which no structure has been determined at present, is a class III nucleotide cyclase domain that is also found in mammalian membrane-bound guanylate and adenylate cyclases.

**Results:**

We have determined the crystal structure of the catalytic domain of a soluble guanylate cyclase from the green algae *Chlamydomonas reinhardtii *at 2.55 Å resolution, and show that it is a dimeric molecule.

**Conclusion:**

Comparison of the structure of the guanylate cyclase domain with the known structures of adenylate cyclases confirms the close similarity in architecture between these two enzymes, as expected from their sequence similarity. The comparison also suggests that the crystallized guanylate cyclase is in an inactive conformation, and the structure provides indications as to how activation might occur. We demonstrate that the two active sites in the dimer exhibit positive cooperativity, with a Hill coefficient of ~1.5. Positive cooperativity has also been observed in the homodimeric mammalian membrane-bound guanylate cyclases. The structure described here provides a reliable model for functional analysis of mammalian guanylate cyclases, which are closely related in sequence.

## Background

The second messenger 3',5'-cyclic guanosine monophosphate (cGMP) is central to many signal transduction pathways, primarily eliciting effects by modulating the activities of phosphodiesterases, protein kinases, and ion channels [1-3]. In mammals, cGMP is synthesized by two distinct classes of guanylate cyclases, which are either cytoplasmic or membrane-bound [4]. Both classes of guanylate cyclase share a catalytic module that is closely related in sequence to that of mammalian adenylate cyclases. The catalytic domain is a class III nucleotide cyclase domain [5], which is distributed widely from bacteria to humans. The class III nucleotide cyclase domain is often found fused to diverse regulatory domains, but is also found as an isolated protein [6-8]. The mammalian membrane-bound guanylate cyclases, which respond to extracellular peptide binding or to the levels of intracellular Ca^2+^, function in maintenance of fluid homeostasis, inhibition of myocyte hypertrophy, skeletal development, and visual and olfactory signal transduction [9]. The mammalian soluble guanylate cyclases are regulated primarily by binding of nitric oxide (NO), and they modulate a wide range of physiological functions, such as maintenance of vascular tone, platelet aggregation, and neurotransmission [10]. Dysfunction of guanylate cyclase signaling underlies many pathophysiological conditions, ranging from stroke and hypertension to gastrointestinal disease and neurodegeneration [11-13].

Mammalian soluble guanylate cyclases are heme-containing heterodimers of homologous α and β subunits [10]. The N-terminal regulatory domain of each subunit contains a heme-NO and/or oxygen-binding (H-NOX) domain [14,15], and the H-NOX domains of the β subunits have been shown to bind the heme cofactor [16,17]. The homologous regions of the α subunits do not bind heme, but are predicted to possess a similar fold. The α and β subunits each contain a central region, shown to be involved in heterodimerization, that consists of an H-NOXA (H-NOX associated) domain and an amphipathic helical extension predicted to form a coiled-coil [18,19]. The catalytic domain is located in the C-terminal segment of the protein, and it associates with the catalytic domain of the partner subunit to form a heterodimeric catalytic unit [20,21]. The mechanism of soluble guanylate cyclase activation by NO involves the binding of NO to the heme cofactor [22], but the details of this activation mechanism are unknown. The response of soluble guanylate cyclase to NO is regulated allosterically by nucleotides [23,24], but how this happens is also not understood.

The three-dimensional structure of a guanylate cyclase catalytic domain has not been reported. Crystal structures have been obtained for an oxygen-bound H-NOX domain of a methyl-accepting chemotaxis protein from the obligate anaerobe *Thermoanaerobacter tencongensis *[25,26] and for NO- and carbon monoxide-bound forms of an H-NOX domain from a histidine kinase operon in the cyanobacterium *Nostoc sp. *[27], yielding clues to the mechanism of soluble guanylate cyclase heme ligand recognition and discrimination. Additionally, the crystal structure of the H-NOXA domain of a signal-transduction histidine kinase from *Nostoc punctiforme *was reported recently [28], revealing that the dimeric H-NOXA domain adopts a Per/Arnt/Sim (PAS) fold and suggesting a mechanism for the preferential heterodimerization exhibited by mammalian soluble guanylate cyclase. Homology modeling based on crystal structures of the related mammalian and bacterial class III adenylate cyclase catalytic domain dimers has provided some information concerning the structure of the catalytic domain of the guanylate cyclases (reviewed in [7]).

**Figure 1:**
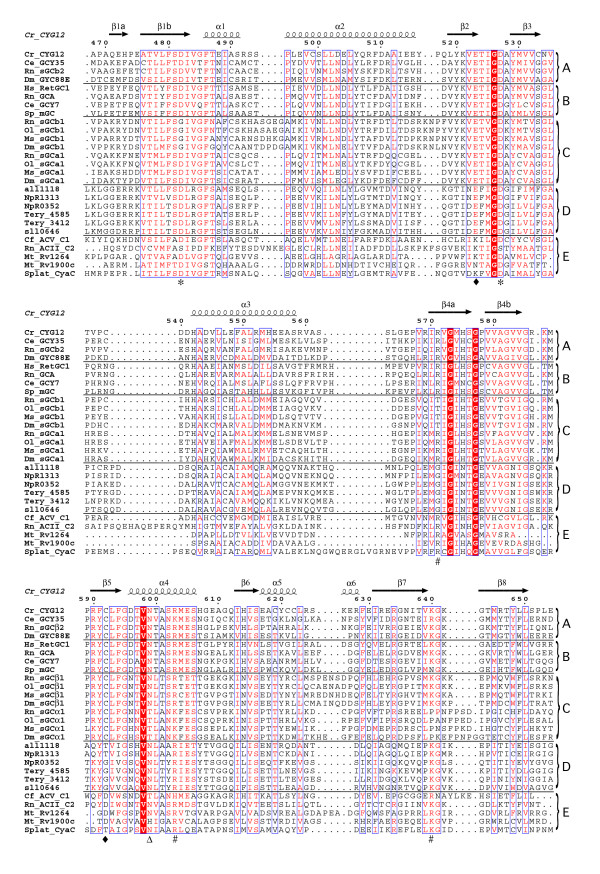
**Structural alignment of selected guanylate and adenylate cyclase catalytic domains**. Secondary structure annotation and numbering correspond to the guanylate cyclase homolog CYG12 from *C. reinhardtii*. Sequences are grouped as follows: A, atypical soluble guanylate cyclases; B, membrane-bound guanylate cyclases; C, NO-sensing soluble guanylate cyclases; D, putative bacterial guanylate cyclases; E, mammalian and bacterial adenylate cyclases. Functional residues are indicated by symbols: metal binding (*); ribose binding (Δ); guanine/adenine binding (◆); triphosphate binding (*#*). Accession numbers are as follows: *Chlamydomonas reinhardtii *CYG12 (GenBank XP_001700847), *Caenorhabditis elegans *GCY35 (GenBank O02298), *Rattus norvegicus *sGCβ2 (GenBank BAB68564), *Drosophila melanogaster *GYC-88E (GenBank Q8INF0), *Homo sapiens *RetGC1 (GenBank Q02846), *R. norvegicus *GCA (GenBank P18910), *C. elegans *GCY7 (GenBank AAQ62451), *Strongylocentrotus purpuratus *mGC (GenBank P16065), *R. norvegicus *sGCβ1 (GenBank BAC55087), *Oryzias latipes *sGCβ1 (GenBank BAA76691), *Manduca sexta *sGCβ1 (GenBank AAC61264), *D. melanogaster *sGCβ1 (GenBank), *R. norvegicus *sGCα1 (GenBank AAB17953), *O. latipes *sGCα1 (GenBank BAA76690), *M. sexta *sGCα1 (GenBank AAC61263), *D. melanogaster *sGCα1 (GenBank AAF56917), *Anabaena sp. *PCC7120 all1118 (GenBank NP_485161), *Nostoc punctiforme *PCC73102 NpR1313 (GenBank YP_001864972), *N. punctiforme *PCC73102 NpR0352 (GenBank ACC79135), *Trichodesmium erythraeum *IMS101 Tery_4585 (GenBank ABG53561), *T. erythraeum *IMS101 Tery_3412 (GenBank ABG52512), *Synechocystis sp. *PCC6803 sll0646 (GenBank BAA16969), *Canis familiaris *ACV_C1 (GenBank 1CJU_A), *R. norvegicus *ACII_C2 (GenBank 1CJU_B), *Mycobacterium tuberculosis *Rv1264 (GenBank 1Y11_A), *M. tuberculosis *Rv1900c (GenBank 1YBU_C), *Spirulina platensis *CyaC (GenBank 1WC1_C). Initial alignments were carried out using the program ClustalX [67]. Sequences were adjusted manually with comparison to results from a structural homology search using the DALI server [45]. Figure 1 was prepared using the program ESPRIPT [68]. Regions containing residues of > 70% equivalence (red letters) are boxed with a thin blue line, and absolutely conserved residues are highlighted in red.

There are distinct soluble guanylate cyclases in invertebrates, including insects, nematodes, and algae [25,29,30]. Also called atypical soluble guanylate cyclases, several of these have been predicted to function as homodimers instead of heterodimers, and a number have been demonstrated to be regulated by oxygen [31-34]. The core subunit architecture outlined above for the mammalian enzymes is conserved in these atypical ones, with additional domains of unknown function appended to the C-terminus of some of the proteins. Here, we report the structure of the catalytic domain of a soluble guanylate cyclase (CYG12) from the unicellular green algae *Chlamydomonas reinhardtii*, which shares 40 to 50% identity with the soluble and membrane-bound guanylate cyclase catalytic domains (Figure 1). The 991-residue full-length CYG12 protein contains each of the domains present in well-characterized soluble guanylate cyclases as well as an additional C-terminal domain of unknown function, and has the full complement of residues necessary to function as a homodimer. As expected, the *C. reinhardtii *guanylate cyclase catalytic domain has the same protein fold as the mammalian adenylate cyclases. With minor differences, the positions of the residues necessary for catalysis and nucleotide base recognition are in the same locations for guanylate and adenylate cyclases, although the identities of the base recognition residues are obviously different. Compared to mammalian adenylate cyclase, the crystal structure is in an inactive conformation, with distorted active site structural elements. Based on the structure, we propose a mechanism for the positive cooperativity that is observed for mammalian homodimeric membrane-bound guanylate cyclases and demonstrated by us for the *C. reinhardtii *guanylate cyclase. We speculate that the activation mechanism for the guanylate cyclases involves structural rearrangement analogous to that exhibited by the mammalian adenylate cyclases.

## Results and discussion

### Structure Determination

We have determined the structure of a *C. reinhardtii *soluble guanylate cyclase catalytic domain dimer by molecular replacement, using the structure of the mammalian adenylate cyclase heterodimer [35] as a search model. The structure contains one catalytic domain dimer per asymmetric unit. During refinement, we observed unexplained peaks in electron density maps around five of the fourteen cysteine residues in the dimer (Cys 499 and Cys 592 of both monomers, and Cys 621 of monomer B). High concentrations of reductant [5–10 mM dithiothreitol and 10 mM tris(2-carboxyethyl)phosphine] were present during crystallization, making it unlikely that cysteine oxidation or disulfide bond formation are responsible for these features. We wondered whether the apparent modification of the cysteine sidechains might be due to the addition of dimethylarsenic groups via reaction of the cysteine thiol group with the sodium cacodylate [sodium dimethylarsenate, (CH_3_)_2_AsO_2_Na] buffer and dithiothreitol reductant present during crystallization [36]. This chemical modification has been observed previously for several proteins crystallized from solutions containing this buffer [37,38]; it has even been used to obtain experimental phases for protein structure solution [39]. Accordingly, we confirmed the presence and location of the dimethylarsenic-modified cysteines by taking advantage of the arsenic anomalous signal to calculate an anomalous difference map, which showed unambiguous peaks of electron density around the dimethylarsenic-modified cysteines (see Additional File 1: Dimethylarsenic cysteine modifications).

The three C-terminal residues of each monomer are disordered, as are residues 564–566 in monomer A and residues 562–565 in monomer B. The final model of the guanylate cyclase dimer includes residues 467–563 and 567–653 for monomer A and residues 467–561 and 566–653 for monomer B. The model also includes 8 phosphate ions and 99 solvent molecules, and was refined to 2.55 Å resolution. After refinement, the final model had working and free R-values [40] of 17.2% and 21.5%, respectively (Table 1).

**Table 1:** Crystallographic data and refinement statistics

**Data collection**	
Beamline	ALS 8.2.2
Wavelength (Å)	1.000
Space group	P3_2_21
Unit cell	*a *= 123.7, *b *= 123.7, *c *= 62. 8 α = 90, β = 90, γ = 120
Resolution (Å)	28-2.55 (2.7-2.55)
R_merge _(%)	7.4 (52.1)
I/σ (I)	7.6 (1.3)
Completeness (%)	96.2 (98.2)
Redundancy	4.3 (4.3)
**Refinement**	
Unique reflections	32518
Free R test set (%)	5
R_work_/R_free_	17.2/21.5
Monomers per A. U.	2
No. atoms	2999
Protein	2860
Ligand	40
Solvent	99
r.m.s. deviation, bond lengths (Å)	0.015
r.m.s. deviation, bond angles (Å)	1.476

### The guanylate cyclase fold

**Figure 2:**
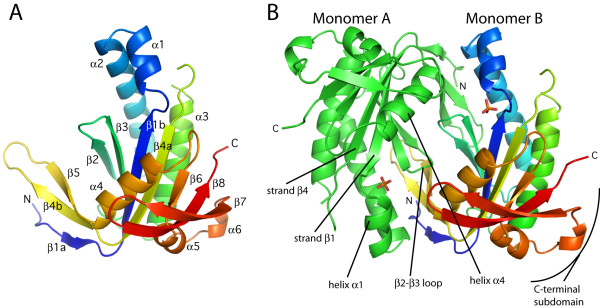
**Structural features of the guanylate cyclase domain**. A) Structural representation of a guanylate cyclase domain monomer. Elements of secondary structure are labeled according to the nomenclature depicted in Figure 1. B) The guanylate cyclase catalytic domain. Monomer A is colored green, and monomer B is multi-colored, ranging from blue at the N-terminus to red at the C-terminus. Two of eight phosphate ions are shown and are depicted as stick figures: phosphorus, orange; oxygen, red.

Each guanylate cyclase domain contains a central seven-stranded β sheet surrounded by several α helices. Secondary structure elements are named according to the convention for adenylate cyclases [35,41] and are indicated in Figure 2a. The first four β strands are part of a βαββαβ arrangement of secondary structure elements, a hallmark of the class III nucleotide cyclase fold, several classes of polymerase, and other nucleotidyltransferases [42-44]. Indeed, a search of the structure database with the program DALI [45] identifies the mammalian adenylate cyclase catalytic domains as the closest structural match, followed by several bacterial adenylate cyclases and polymerases. A smaller 3-stranded β sheet, formed by strand β5 and extensions of β1 and β4 (β1a and β4b), extends from the core of the domain, and interacts with strands β2 and β3 of the opposite monomer to form part of the dimer interface. The two monomers in the wreath-like dimer [41] are related by a twofold axis, and a central cleft, formed by the dimer interface, contains the two symmetric active sites (Figure 2b). The two monomers superimpose on each other with a r.m.s. deviation of 0.3 Å for 160 structurally equivalent Cα atom pairs. The primary structural differences between the monomers are found in their C-terminal subdomains, particularly in the α6–β7 and β7–β8 loops, and are due to differences in crystal packing interactions.

The structure of the guanylate cyclase catalytic domain differs from that of the adenylate cyclases primarily in the elements that connect strands and helices, and in the less-conserved C-terminal subdomains (Figure 1). Biochemical and structural studies have shown that these regions in the adenylate cyclases couple to regulatory proteins such as the heterotrimeric G-protein subunits G_s_α, G_i_α, and protein kinase C (reviewed in [46]). Differences in sequence and structure between individual catalytic subunits in heterodimeric adenylate and guanylate cyclases also localize to the same regions (Figure 1).

### The active site

**Figure 3:**
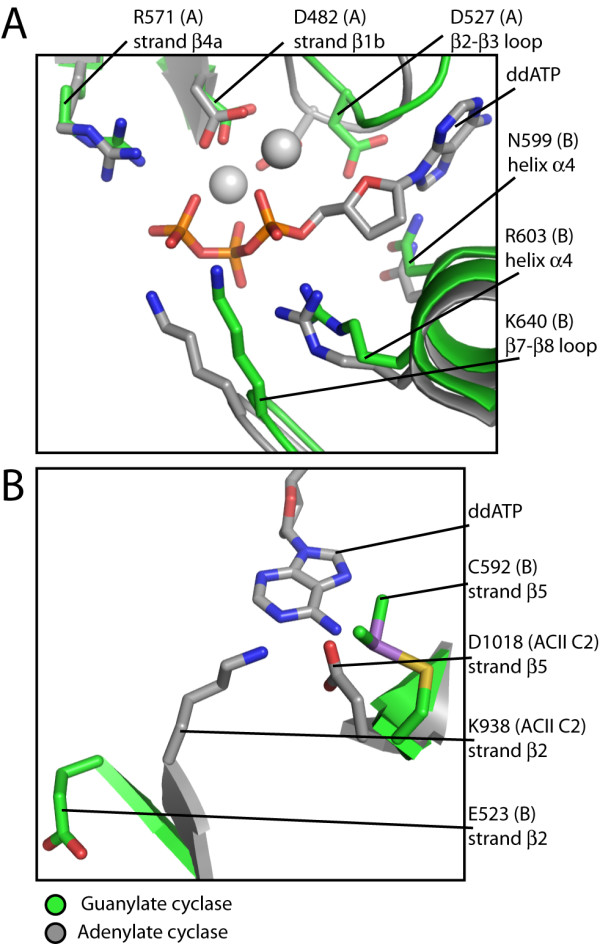
**Comparison between guanylate and adenylate cyclase active sites**. Monomer B of the guanylate cyclase catalytic domain was superimposed onto the C2 domain of mammalian adenylate cyclase (PDB ID: 1CJU) [49]. Residues and structural elements involved in catalysis and nucleotide recognition are shown. A) Comparison of guanylate and adenylate cyclase catalytic residues. B) Comparison of guanylate and adenylate cyclase base recognition residues. Side chains and structural elements from the guanylate cyclase and adenylate cyclase catalytic domains are colored green and grey, respectively. The nucleotide 2',3'-dideoxyadenosine triphosphate (ddATP) and the two Mg^2+ ^ions are from 1CJU. Non-carbon atoms are colored as follows: phosphorus, orange; oxygen, red; nitrogen, blue; sulfur, yellow; arsenic, violet; magnesium, white.

The catalytic residues necessary for synthesizing cyclic nucleotides, conserved across all adenylate cyclases and guanylate cyclases (Figure 1), are contributed by structural elements of both monomers at each active site. We will focus on one active site, and refer to each domain as monomer A or B. In the *C. reinhardtii *guanylate cyclase described here, the catalytic residues are the metal-binding residues Asp 482(A) and Asp 527(A) located in the β2–β3 loop and in strand β1b, respectively, the ribose-orienting residue Asn 599(B) and the transition-state-stabilizing residue Arg 603(B), both located in helixα4, and the triphosphate-positioning residues Arg 571(A) in strandβ4a and Lys 640(B) in the β7–β8 loop (Figure 3a). With the exception of the critical metal-binding residue Asp 527(A) (see below), all of these active site residues are located at positions analogous to their location in the adenylate cyclase active site (Figure 3a); minor conformational differences likely reflect the absence of metals and nucleotides, which would assist in organizing the active site into a catalytically competent conformation.

Modeling based on the adenylate cyclase structures has indicated the mechanism of nucleotide base discrimination [47,48]. A glutamic acid and a cysteine, conserved in guanylate cyclases, have been proposed to mediate recognition of the exocyclic amine and carbonyl group of the guanine base, respectively. The specificity-determining residues in mammalian adenylate cyclase, a lysine and an aspartic acid, are located at the same relative positions in the adenylate cyclase protein sequence. In fact, swapping those residues into a guanylate cyclase catalytic domain results in conversion into an adenylate cyclase [47,48], underscoring the equivalence of the catalytic machinery between guanylate and adenylate cyclases. In our structure, the corresponding residues are Glu 523(B) and Cys 592(B), which are situated close to the locations of their adenylate cyclase counterparts (Figure 3b). Local distortions caused by the dimethylarsenic modifications in each monomer appear to prevent optimal sidechain orientation for base recognition. For Cys 592(B), the dimethylarsenic modification of the thiol side chain prevents potential hydrogen bonding interaction with the exocyclic carbonyl group of a substrate GTP molecule (Figure 3b). The modification also results in distortion of the β2–β3 loop that contains the metal-binding residue Asp 527(A) and the base-recognition residue Glu 523(A), causing it to adopt a conformation incompatible with binding a metal-nucleotide complex (Figure 3a). Together, these local distortions would likely preclude any nucleotide binding in the active site, providing a rationale for our failure to visualize nucleotides and metals that have been soaked into the crystals.

### Guanylate cyclase activation

Activation of the mammalian adenylate cyclase has been proposed to proceed via two steps, based on structures of active and inactive forms of the protein [35,41,49]. In the first step, binding of G_s_α between the α1–α2 and α3–β4 loops of the C2 subunit causes a 7° rotation of the core of the C1 subunit around an axis that runs parallel to the central cleft, priming the active site for catalysis by bringing the catalytic residues from one subunit ~2 Å closer to the catalytic residues of the other [35]. The second step involves the closure of the active site around the bound nucleotide. This closure brings structural elements that bind the metal cofactors and the nucleotide triphosphate moiety, and residues in the opposite subunit that orient the ribose ring and stabilize the transition state, into optimal alignment for catalysis [49] (see Additional file 2: Activation mechanism of mammalian adenylate cyclase). In particular, helix α1 moves towards helix α4 of the opposite subunit, such that a triphosphate interaction site is formed from the helix α1 dipole and a P-loop-like structure between strand β1b and helix α1. The N-terminal end of strand β4, the C-terminal end of strand β1, and the N-termini of helices α2 and α3 also shift towards the active site, properly orienting metal-binding and triphosphate-binding residues for catalysis.

Comparison of our guanylate cyclase catalytic domain structure to structures of the mammalian adenylate cyclase suggests that the guanylate cyclase catalytic domain is in an inactive conformation. The dimethylarsenic modifications described above clearly distort several active site residues. But, in addition to these localized changes, the overall orientation of one subunit with respect to the other corresponds to an open state that must close considerably for catalysis to occur, as we discuss further below.

**Figure 4:**
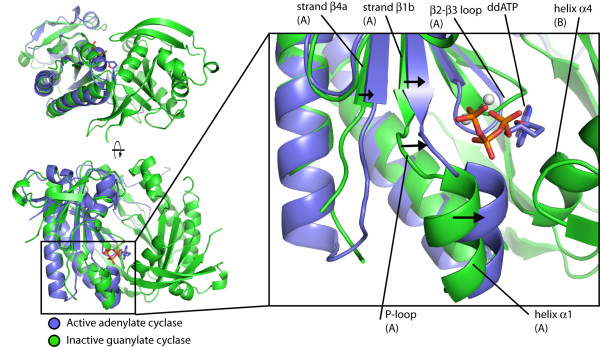
**Proposed guanylate cyclase activation mechanism**. Comparison of helix α1 in the guanylate cyclase structure with helix α1 of the active mammalian adenylate cyclase structure indicates that the guanylate cyclase structure is in an inactive state. Monomer B of the guanylate cyclase structure was superimposed onto the C2 domain of the active adenylate cyclase structure (PDB ID: 1CJU) [49] and monomer A was superimposed onto the C1 domain of the same adenylate cyclase structure. The C2 domain is omitted for clarity. The C1 domain of the active adenylate cyclase structure is colored blue, and the guanylate cyclase structure is colored green. The nucleotide 2',3'-dideoxyadenosine triphosphate (ddATP) and Mg^2+ ^ions from 1CJU are shown as a stick figure and spheres: phosphorus, orange; oxygen, red; nitrogen, blue; magnesium, white.

The signature structural change upon activation for all adenylate cyclases is the movement of helix α1 towards the active site and helix α4 in the opposite subunit, regardless of other changes in domain orientations. In our structure, a phosphate ion is bound to the N-terminal end of helix α1 and to the P-loop-like site, suggesting the presence of a likely triphosphate-coordination site. However, when monomer A of the guanylate cyclase structure is superimposed on the active adenylate cyclase C1 domain, helix α1 has not moved towards the active site, which must occur to properly align all the catalytic residues, bind nucleotide, and achieve an active conformation (Figure 4). Instead, the conformation of helix α1 in the guanylate cyclase structure is much closer to that of helix α1 in the inactive adenylate cyclase structure (compare Figure 4 and Additional file 2: Activation mechanism of mammalian adenylate cyclase). Activation of the guanylate cyclase domains in the structure reported here would require the N-terminus of helix α1 of each monomer to move ~3 Å towards helix α4 of the other monomer, resulting in the concomitant shifting of the ends of strands β1b and β4a inwards towards the opposite monomer, and leading to an active site configuration similar to that observed for the mammalian adenylate cyclases (Figure 4).

### Active site cooperativity

**Figure 5:**
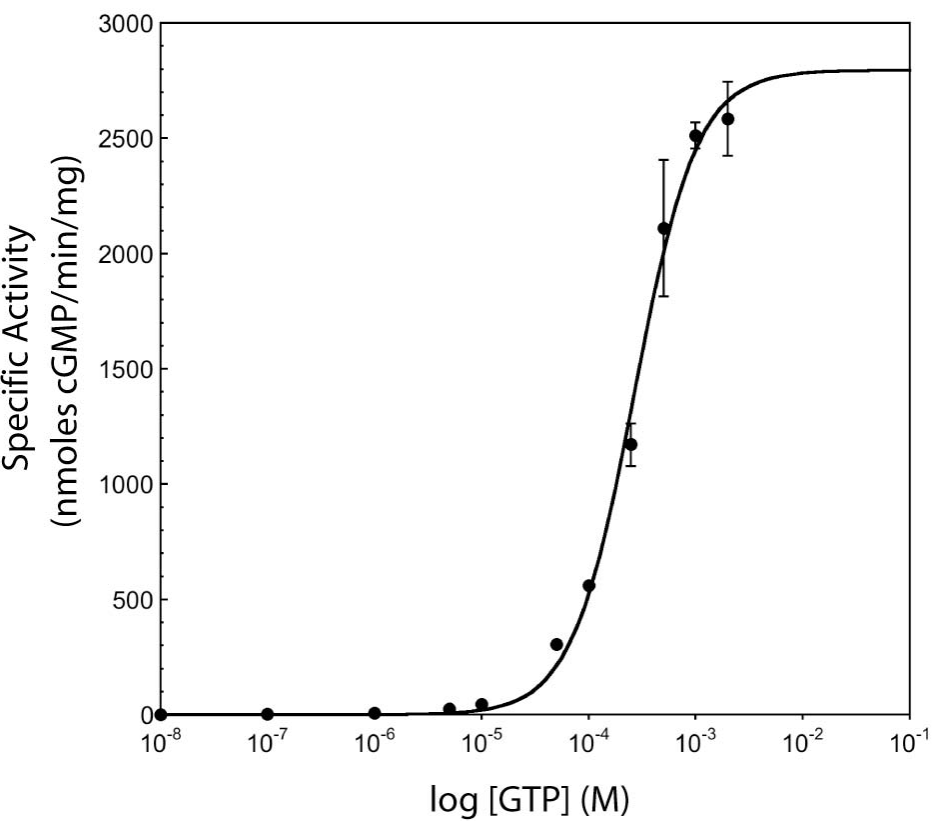
**Communication between active sites**. Plot of guanylate cyclase activity at increasing concentrations of substrate GTP. Guanylate cyclase (5 μg) was incubated for 2 min at 24 °C with the indicated concentrations of GTP in the presence of 4 mM MnCl_2 _and cGMP was measured. Data were fit to the equation $\frac{V_{\max}{\left(S\right)}^{n}}{{\left(S_{0.5}\right)}^{n}+S^{n}}$, where *V*_max _is the maximum activity, *S *is the concentration of GTP, *S*_0.5 _is the substrate concentration at which half-maximal velocity is reached, and *n *is the Hill coefficient. From the fit, *V*_max _= 2795 ± 117 nmoles cGMP/min/mg, *S*_0.5 _= 269 ± 26 μM, and *n *= 1.49 ± 0.16. A Hill coefficient greater than 1 indicates the presence of interacting active sites.

The mammalian adenylate cyclase catalytic domain heterodimer contains one active site and one catalytically non-functional site – each monomer is missing residues required for catalysis, which are provided by the other monomer. The homodimeric nature of the guanylate cyclase catalytic domain described here suggests that it contains two active sites. In fact, the existence of two active sites has been postulated for mammalian membrane guanylate cyclases, all of which function as homodimers [50]. We sought to confirm this possibility by looking for evidence of cooperativity in activity assays. Activity assays were carried out in the presence of Mn^2+^, because the activity in the presence of Mg^2+ ^was less than 1% of that in the presence of Mn^2+ ^(data not shown), as seen for mammalian soluble guanylate cyclase catalytic domains [21]. We found that the enzyme exhibits positive cooperativity, with a Hill coefficient of 1.5 (Figure 5), indicating the presence of more than one active site and providing evidence that the active sites interact with each other. Cooperativity has also been observed for other homodimeric cyclases, such as the mammalian membrane-bound guanylate cyclases [51-53], as well as some bacterial adenylate cyclases [54,55]. A possible mechanism for communication between the mammalian adenylate cyclase active site and the pseudosymmetric site, where the activator forskolin binds, has been proposed. In the mammalian adenylate cyclase catalytic domain heterodimer, the β2–β3 loop of the C1 monomer, which contains a catalytically essential aspartic acid, is connected via a hydrogen bond and a hydrophobic interaction to the β2–β3 loop of the C2 monomer, which forms part of the forskolin binding site. These interactions between the forskolin binding site and the active site provide a possible mechanism by which the binding of forskolin is communicated to the catalytic residues in the active site, resulting in activation [35].

In the absence of nucleotide, it is not clear in our structure how occupation of one active site by nucleotide is detected by the other. The analysis is also complicated by the local distortions near the active site caused by the dimethylarsenic-modified cysteines. However, inspection of our structure provides two possible mechanisms for communication between the active sites. The first mechanism involves a direct connection between residues in the two active sites. The β2–β3 loop of each monomer carries both the invariant catalytic residue Asp 527 for one active site and the conserved guanine-binding residue Glu 523 for the opposite active site. We propose that the interaction of either Asp 527 or Glu 523 with nucleotide in one active site could alter the conformation of the loop in which they both reside, resulting in a change in nucleotide affinity or enhanced catalysis in the other active site. The second mechanism involves propagation of local changes in one active site, through changes in elements of secondary structure, to the other active site. As noted above, upon substrate binding, helix α1 and strands β1 and β4 in monomer A move inward towards crucial residues in helix α4 of monomer B, bringing residues from both monomers into correct alignment for catalysis in one active site (Figure 4). This movement also brings helix α4 of monomer A, which lies directly above, and packs against, strands β1 and β4 of monomer A, towards monomer B. As helix α4 of monomer A carries residues necessary for catalysis at the other active site, this movement might allow nucleotide binding at one active site to effectively begin pre-organizing the other active site for nucleotide binding.

### Interaction with regulators

**Figure 6:**
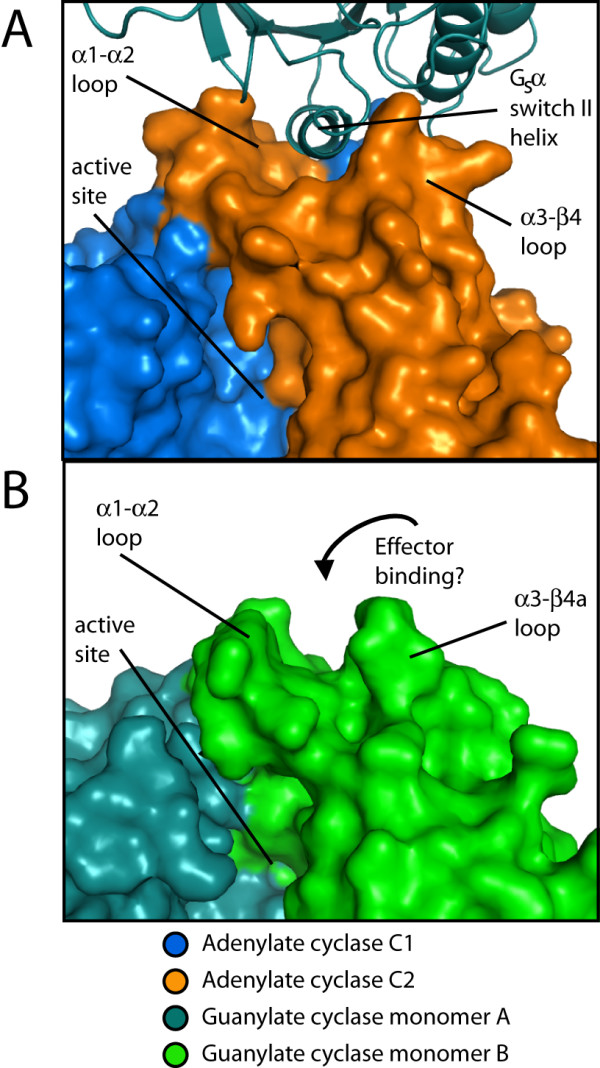
**A potential binding site for a regulatory control element**. A) Structure of the mammalian adenylate cyclase catalytic domain bound to the activator G_s_α (PDB ID 1CJU) [49]. The switch II helix of G_s_α binds in a groove on the C2 domain between the α1–α2 and α3-β4a loops, priming the catalytic domain for nucleotide binding. A surface representation of the adenylate cyclase catalytic domain is shown, and G_s_α is shown as a ribbon cartoon. The C1 domain is colored blue, the C2 domain is colored orange, and G_s_α is colored teal. B) Surface representation of the guanylate cyclase catalytic domain in the same orientation as the adenylate cyclase domain in A. A groove similar to that used by adenylate cyclase to bind to G_s_α is located between the α1–α2 and α3-β4a loops, and may serve as a site for interaction of control elements with the guanylate cyclase catalytic domain. Monomer A is colored teal, and monomer B is colored green.

The activating conformational transition of helix α1 and attendant shift in adjacent β strands might be facilitated by a domain rotation like that observed for the mammalian adenylate cyclase C1 monomer upon G_s_α binding [35]. In the adenylate cyclases, the α1–α2 and α3–β4 loops of the C2 domain form a groove into which the switch II helix of G_s_α is docked. The docking of G_s_α brings about the rotation of the C1 domain. A similar groove is found in the analogous location on the guanylate cyclase dimer structure (Figure 6). It is tempting to speculate that some regulatory element, such as a soluble guanylate cyclase H-NOX sensor domain, might interact with this region in an analogous fashion, altering the balance of conformations in the catalytic domain. It is also possible that interaction of regulators with entirely different structural elements, such as the C-terminal subdomains, may be required to activate the guanylate cyclase catalytic domain. Answers to such questions await the structure of an active guanylate cyclase domain in the presence of regulatory elements.

## Conclusion

We report the first structure of a eukaryotic guanylate cyclase catalytic domain. The resemblance of the domain to that of the mammalian adenylate cyclase is unsurprising, given the sequence and functional similarity between them. Nevertheless, more than ten years have elapsed between the first reports of the structures of the adenylate cyclases [35,41] and our results, presented here. The difficulty in crystallizing a guanylate cyclase domain may reflect an increased intrinsic flexibility in the guanylate cyclase domain relative to the adenylate cyclase domain, and it is possible that we succeeded in part because of the fortuitous cysteine modifications that may have increased the rigidity of the domain, facilitating crystallization. We have been unable as yet to crystallize the catalytic domain in the absence of these modifications.

The high degree of sequence conservation between the soluble guanylate cyclase catalytic domain described here and the catalytic domains of mammalian soluble and membrane-bound guanylate cyclases (40 to 50% identity) suggests that our structure will serve as a superior model for functional studies, compared to the mammalian adenylate cyclase catalytic domains (25 to 30% sequence identity). Our structure indicates that the differences between the adenylate and guanylate cyclase are generally localized to flexible regions, some of which are proposed to mediate coupling with regulatory domains and other control elements. While specific differences in regulatory interactions are likely determined by the sequence and local structure of these variable elements, the overall activation mechanism, involving conformational switching by helix α1 and attendant changes in the adjacent β sheet, is expected to be conserved.

## Methods

### Cloning and protein purification

PCR was used to amplify the gene encoding a 991-residue soluble guanylate cyclase homolog CYG12 (GenBank: XM_001700795) from a *C. reinhardtii *cDNA library obtained from the Chlamydomonas Center [56]. Forward and reverse PCR primers were 5'-ATGCTGGGCTGGTATGACCGT-3' and 5'-TTACTCCAAACACGGGTTGTCA-3', respectively. PCR products were phosphorylated, blunt-cloned into the vector pGEM, and verified by sequencing (UC Berkeley DNA Sequencing Facility). The guanylate cyclase catalytic domain was expressed and purified using a SUMO-based system (LifeSensors) as follows: residues 468–655, which comprise the catalytic domain of CYG12, were subcloned into a vector containing the yeast SUMO homolog SMT3 with an N-terminal His-tag, and the fusion protein was expressed in *Escherichia coli *Tuner(DE3) (Invitrogen) for 18 h at 20°C. The fusion protein was purified from supernatant by passage over a HisTrap Ni Sepharose affinity column (GE Healthcare). A His-tagged version of the SMT3-specific protease was used to cleave the N-terminal SMT3 fusion partner from the guanylate cyclase domain, which was separated from the protease and SMT3 by a second Ni affinity step. The guanylate cyclase domain was further purified by Q Sepharose anion-exchange chromatography, followed by gel filtration into a final buffer of 25 mM triethanolamine, pH 7.5, 25 mM NaCl, and 10 mM dithiothreitol. Purified protein was concentrated and stored at -20°C until use. Protein concentrations were determined by absorbance using the calculated extinction coefficient ε_280 _= 7680 M^-1 ^cm^-1^.

### Crystallization and X-ray data collection

Crystals were grown using the sitting-drop vapor diffusion method. Equal volumes (200 nl) of protein [40–60 mg/ml in 25 mM triethanolamine, pH 7.5, 25 mM NaCl, 5–10 mM dithiothreitol, 10 mM tris(2-carboxyethyl)phosphine] were mixed with crystallization solution [0.1 M sodium cacodylate, pH 5.0–6.4, 42–62% saturated (NH_4_)_2_HPO_4_] and then equilibrated with a 100-μl reservoir of the same crystallization buffer at 20°C. Crystals grew in the trigonal space group and appeared within 1–2 days. Crystals were transferred to a solution of mother liquor containing 28% glycerol as a cryoprotectant, and cryo-cooled and stored in liquid nitrogen. Diffraction data were collected at 100 K using synchrotron radiation at beam line 8.2.2 at the Advanced Light Source, Lawrence Berkeley National Laboratory. Reflections were integrated and scaled with the programs MOSFLM [57] and SCALA [58]. The structure was solved by molecular replacement with the program PHASER [59] using the mammalian adenylate cyclase catalytic domain (PDB code 1AZS) [35] as the search model, and the space group was identified as P3_2_21. Map improvement was carried out using ARP/wARP [60] and RESOLVE [61]. The model was built using COOT [62] and refined using PHENIX [63]. Six TLS domains were used during refinement: residues 467–475 and 578–595, monomer A/B; residues 476–560, 569–577, and 596–608, monomer A/B; and residues 613–651, monomer A/B. Analysis of model quality was carried out using MOLPROBITY [64]. Figures were prepared using PYMOL [65]. The atomic coordinates and structure factors have been deposited in the Protein Data Bank (3ET6).

### Guanylate cyclase assays

Guanylate cyclase assays were performed in duplicate at 24°C as described previously [66]. Assays contained 5 μg of guanylate cyclase in 25 mM triethanolamine, pH 7.5, 25 mM NaCl, 4 mM MnCl_2 _or MgCl_2 _and 5 mM dithiothreitol. Assays were initiated by addition of indicated amounts of GTP, and were quenched after 2 minutes by addition of 400 μl of 125 mM Zn(CH_3_CO_2_)_2 _and 500 μl of 125 mM Na_2_CO_3_. cGMP was quantified using a cGMP enzyme immunoassay kit, format B (Biomol), per the manufacturer's instructions. Experiments were repeated three times to ensure reproducibility.

## Authors' contributions

JAW designed and performed research, analyzed data, and drafted the manuscript. ERD performed research and analyzed data. MHL analyzed data. MAM designed research and analyzed data. JK designed research, analyzed data, and drafted the manuscript. All authors read and approved the final manuscript.

## Supplementary Material

Additional file 1 **Dimethylarsenic cysteine modifications**. Five cysteine residues are modified through a reaction with the sodium cacodylate and dithiothreitol in the crystallization buffer, resulting in addition of dimethylarsenic to the cysteine thiols. Experimental anomalous difference density contoured at 5 σ is shown superimposed onto the refined guanylate cyclase structure. Sidechains are show as stick figures and colored as follows: carbon, green; sulfur, yellow; arsenic, violet. Click here for file

Additional file 2 **Activation mechanism of mammalian adenylate cyclase**. Helix α1 in the C1 domain of mammalian adenylate cyclase undergoes a significant conformational change going from an inactive structure to an active structure. A hypothetical inactive structure of the mammalian adenylate cyclase catalytic domain was generated as previously described [35]: the C2 domain structure from 1AZS was superimposed on chain A from the structure of a C2 domain homodimer (PDB ID: 1AB8) [41], and the C1 domain structure from 1AZS was superimposed on chain B from 1AB8. The nucleotide- and G_s_α-bound active C1/C2 dimer structure 1CJU [49] was then superimposed onto the C2 domain of the inactive model. The inactive model is colored white, and the active structure is colored blue. G_s_α and the C2 domain of 1CJU are omitted for clarity. The nucleotide 2',3'-dideoxyadenosine triphosphate (ddATP) and Mg^2+ ^ions from 1CJU are shown as a stick figure and spheres: phosphorus, orange; oxygen, red; nitrogen, blue; magnesium, white. Click here for file
