# Supplementary figures and images for: The crystal structure of the catalytic domain of a eukaryotic guanylate cyclase
Source: BMC Struct Biol. 2008 Oct 7;8:42. doi: 10.1186/1472-6807-8-42 (PMC2576301; doi:10.1186/1472-6807-8-42)

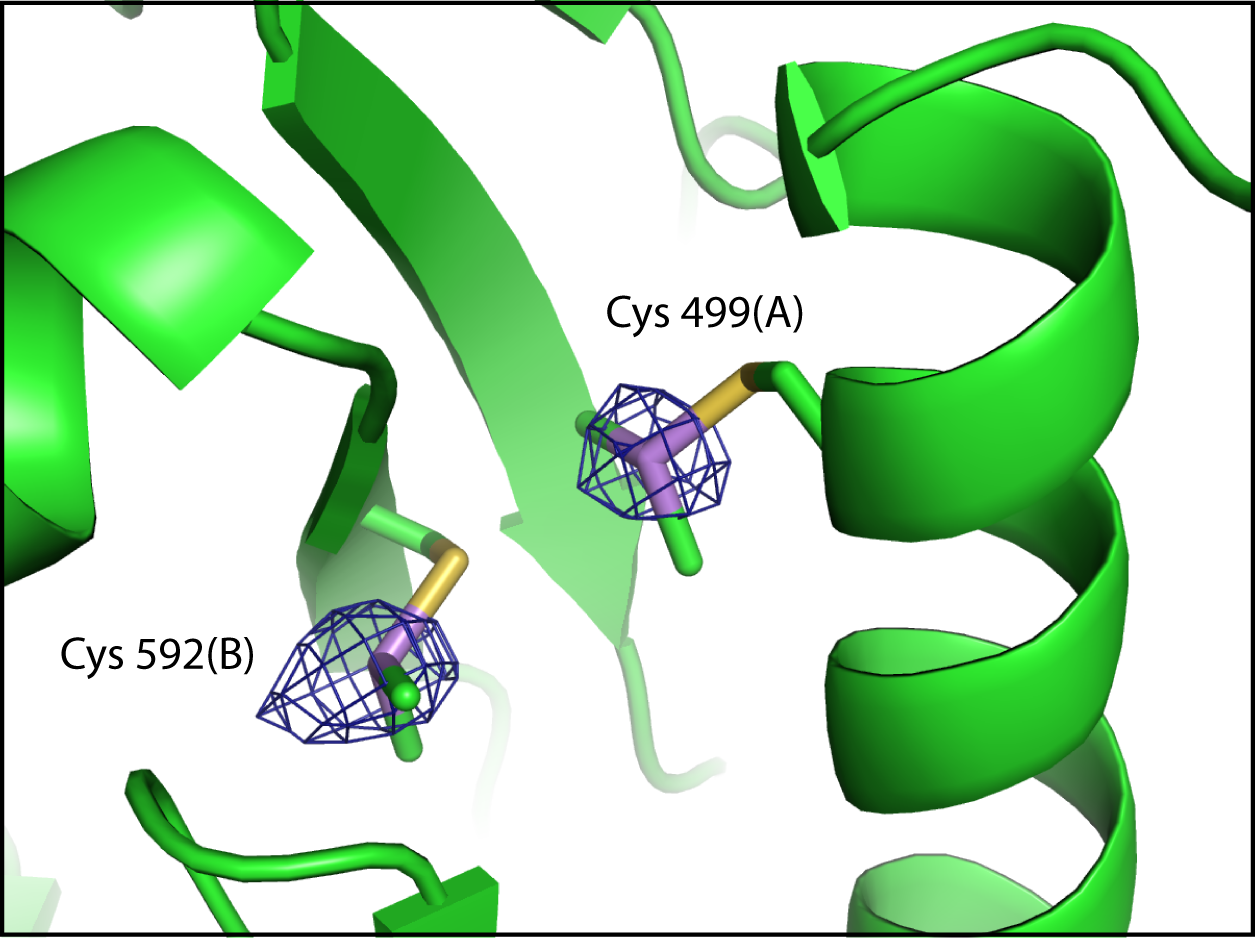

Supplement: Additional file 1 — Dimethylarsenic cysteine modifications. Five cysteine residues are modified through a reaction with the sodium cacodylate and dithiothreitol in the crystallization buffer, resulting in addition of dimethylarsenic to the cysteine thiols. Experimental anomalous difference density contoured at 5 σ is shown superimposed onto the refined guanylate cyclase structure. Sidechains are show as stick figures and colored as follows: carbon, green; sulfur, yellow; arsenic, violet. [file 1472-6807-8-42-S1.png]

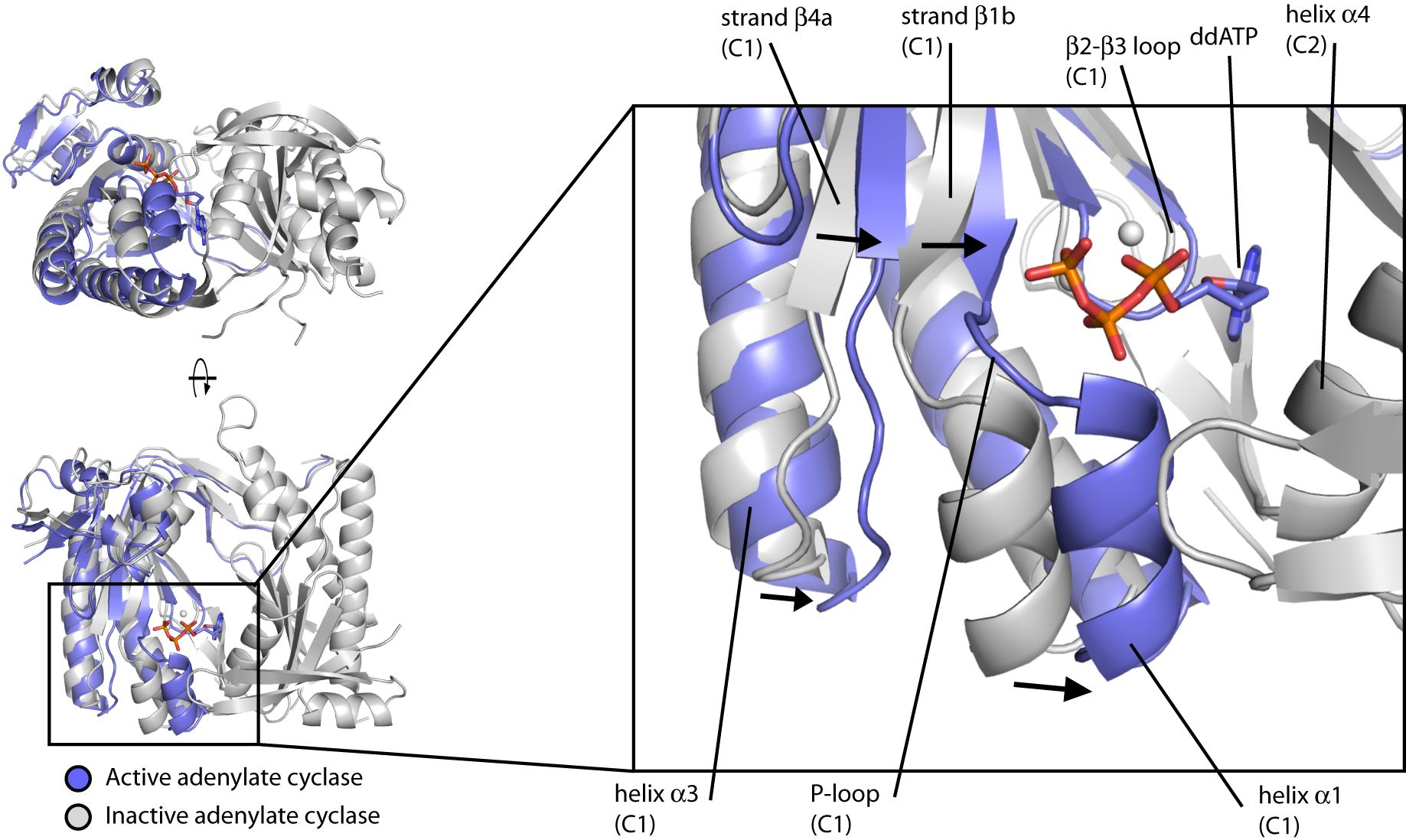

Supplement: Additional file 2 — Activation mechanism of mammalian adenylate cyclase. Helix α1 in the C1 domain of mammalian adenylate cyclase undergoes a significant conformational change going from an inactive structure to an active structure. A hypothetical inactive structure of the mammalian adenylate cyclase catalytic domain was generated as previously described [35]: the C2 domain structure from 1AZS was superimposed on chain A from the structure of a C2 domain homodimer (PDB ID: 1AB8) [41], and the C1 domain structure from 1AZS was superimposed on chain B from 1AB8. The nucleotide- and Gsα-bound active C1/C2 dimer structure 1CJU [49] was then superimposed onto the C2 domain of the inactive model. The inactive model is colored white, and the active structure is colored blue. Gsα and the C2 domain of 1CJU are omitted for clarity. The nucleotide 2',3'-dideoxyadenosine triphosphate (ddATP) and Mg2+ ions from 1CJU are shown as a stick figure and spheres: phosphorus, orange; oxygen, red; nitrogen, blue; magnesium, white. [file 1472-6807-8-42-S2.png]
